# Supplementary figures and images for: A pals-25 gain-of-function allele triggers systemic resistance against natural pathogens of C. elegans
Source: PLoS Genet. 2022 Oct 3;18(10):e1010314. doi: 10.1371/journal.pgen.1010314 (PMC9560605; doi:10.1371/journal.pgen.1010314)

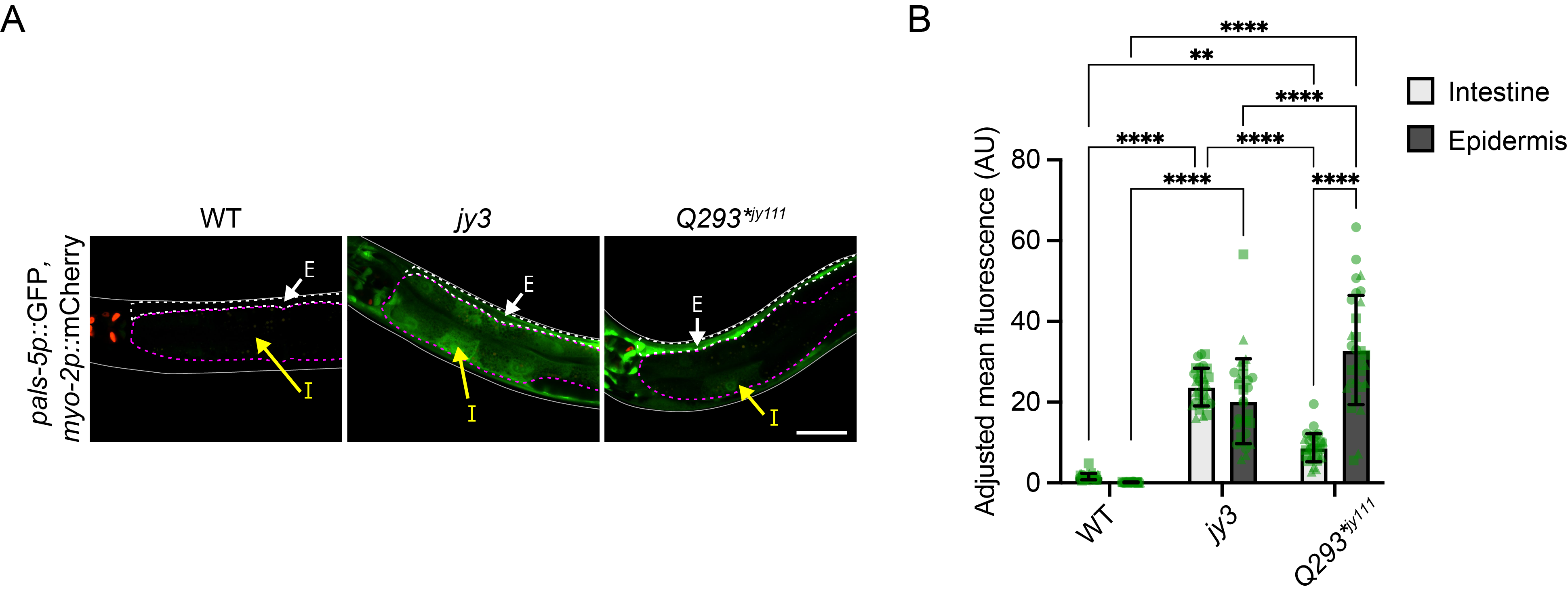

Supplement: S1 Fig — A) Representative images of WT, pals-22(jy3), and pals-25(Q293*)jy111 L4 animals expressing the pals-5p::GFP IPR reporter. Solid grey lines denote the outline of the body, dashed magenta lines outline the anterior intestine, and dashed white lines outline one lateral side of the epidermis adjacent to the intestine. Yellow arrows indicate intestinal tissue (I) and white arrows indicate epidermal tissue (E). Scale bar = 20 μm. B) Quantification of the intestinal and epidermal regions-of-interest shown in A. pals-22(jy3) mutants display similar degrees of pals-5p::GFP expression in the intestine and epidermis. By contrast, pals-25(Q293*)jy111 mutants display stronger pals-5p::GFP in the epidermis, but weaker expression in the intestine, when compared to pals-22(jy3) mutants. **** p < 0.0001, ** p < 0.01, two-way ANOVA with Sidak’s multiple comparisons test. n = 30 animals per genotype analyzed for both intestinal and epidermal expression across three experimental replicates. Different symbol shapes represent animals imaged on different days. (TIF) [file pgen.1010314.s001.tif]

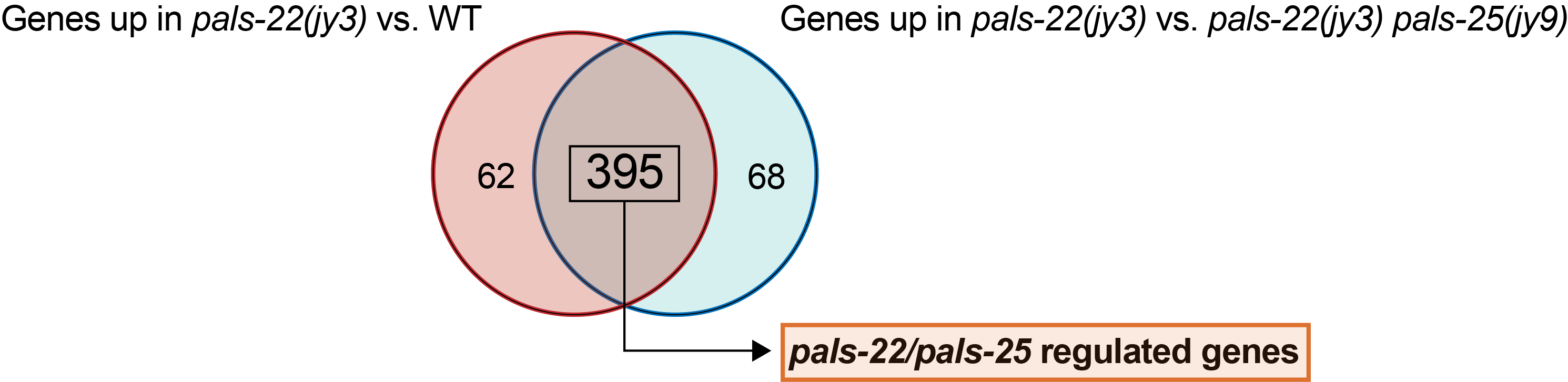

Supplement: S2 Fig — Venn diagram of differentially expressed genes in pals-22(jy3) mutants as compared to wild-type animals, and pals-22(jy3) mutants as compared to pals-22(jy3) pals-25(jy9) double mutants. The overlap of these differentially expressed gene sets is significant and represents genes regulated by pals-22 and pals-25. Hypergeometric test, RF = 26.9; p < 0.000e-00. (TIF) [file pgen.1010314.s002.tif]

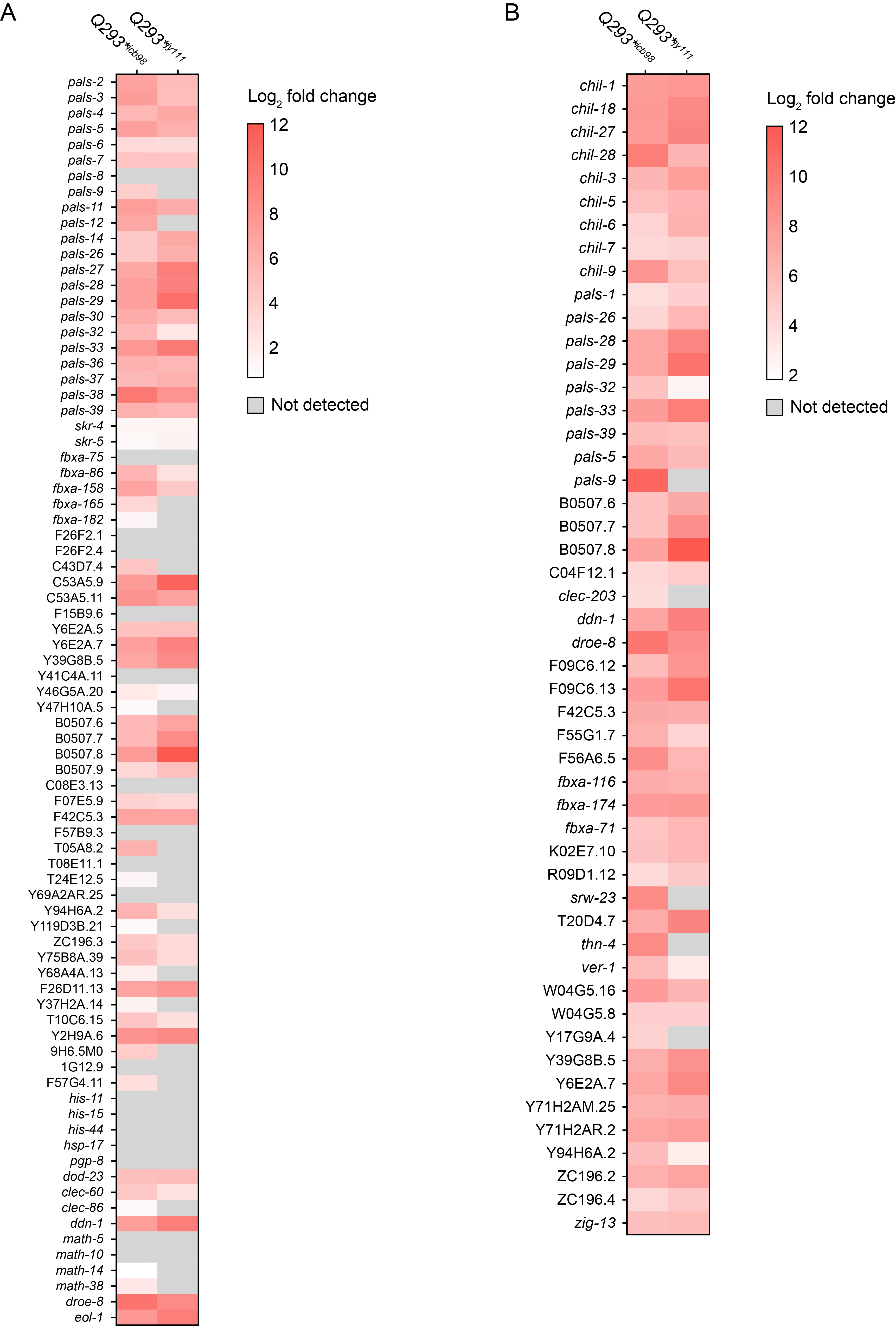

Supplement: S3 Fig — Heatmap of pals-25(Q293*)icb98 and pals-25(Q293*)jy111 mutant gene expression values measured as log2 fold change compared to the 80 genes of the IPR (A) and 50 commonly upregulated genes of the ORR (B). Several genes, for example pals-26, belong to both the IPR and ORR. For comparisons to the IPR in A, some log2 fold change values shown for pals-25(Q293*)icb98 and pals-25(Q293*)jy111 mutants are less than 2 for consistency with the previously published Reddy et al. 2019 IPR dataset. Genes where transcripts were not detected, or abundances were below cutoff levels during analysis (Materials and Methods), are shown in grey. (TIF) [file pgen.1010314.s003.tif]

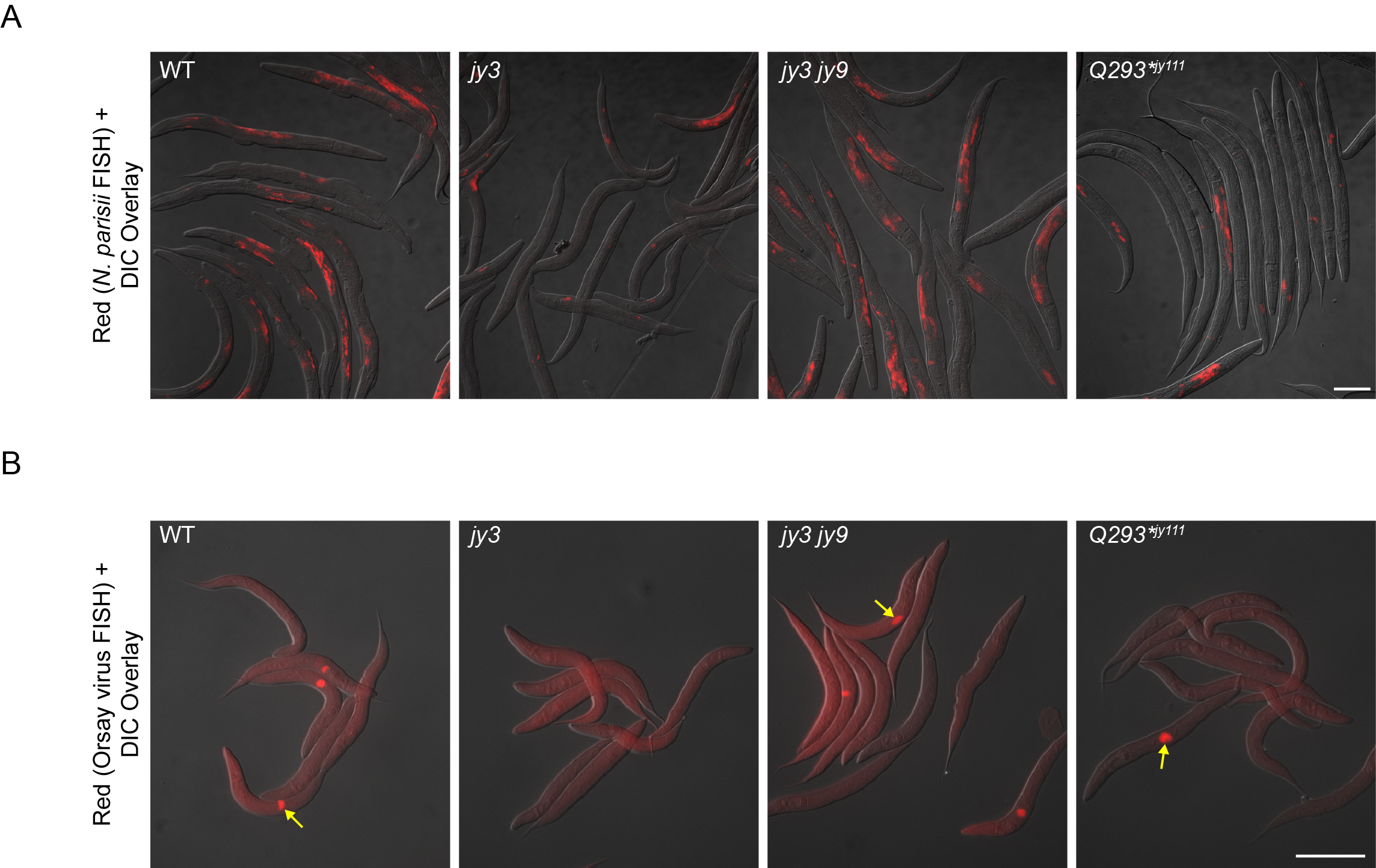

Supplement: S4 Fig — A) Representative images of WT animals, pals-22(jy3), pals-22(jy3) pals-25(jy9), and pals-25(Q293*)jy111 mutants infected at L1 with N. parisii, fixed at 30 hpi, and stained with N. parisii-specific FISH probe (red fluorescence). B) Representative images of WT animals, pals-22(jy3), pals-22(jy3) pals-25(jy9), and pals-25(Q293*)jy111 mutants infected at L1 with Orsay virus, fixed at 18 hpi, and stained with Orsay virus-specific FISH probe (red fluorescence representing viral infection denoted by yellow arrows). For A, B scale bar = 100 μm. DIC = differential interference contrast. (TIF) [file pgen.1010314.s004.tif]

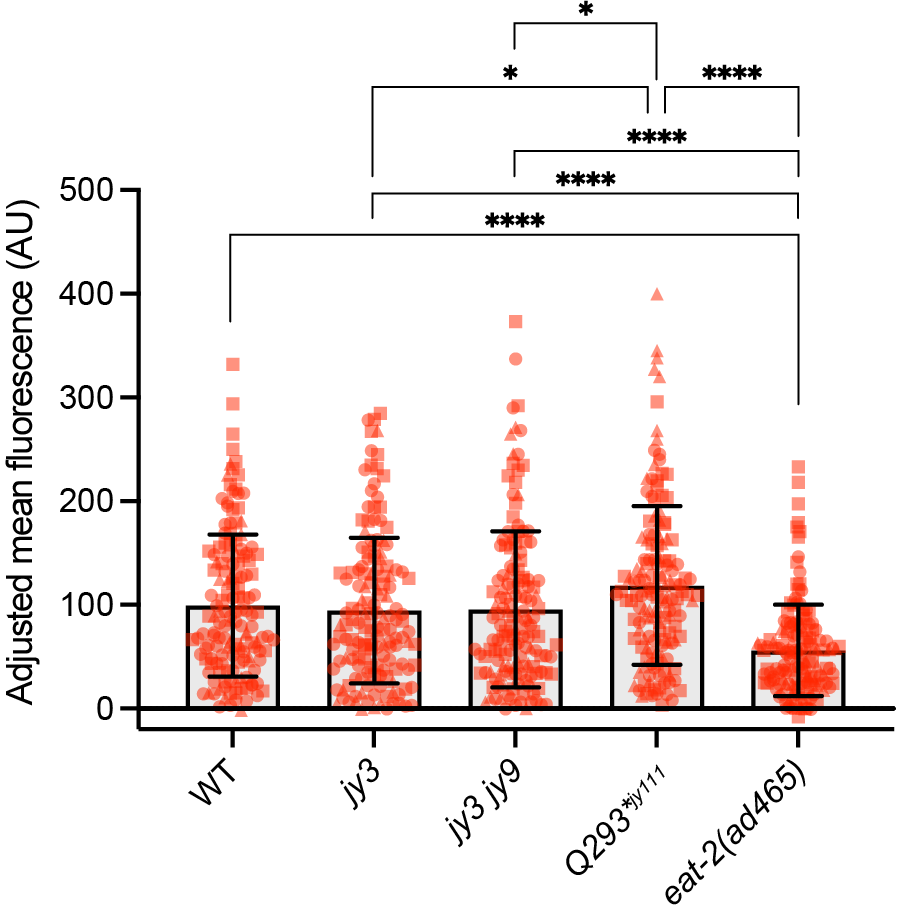

Supplement: S5 Fig — Quantification of fluorescent bead accumulation in WT animals, pals-22(jy3), pals-22(jy3) pals-25(jy9), and pals-25(Q293*)jy111 mutants. eat-2(ad465) mutants have a known feeding defect due to abnormal pharyngal pumping and were used as a positive control. **** p < 0.0001, * p < 0.05, Kruskal-Wallis test with Dunn’s multiple comparisons test. n = 150 animals per genotype, three experimental replicates. Symbols represent fluorescence measurements for individual animals and different symbol shapes represent animals from experimental replicates performed on different days. Bar heights indicate mean values and error bars represent standard deviations. (TIF) [file pgen.1010314.s005.tif]

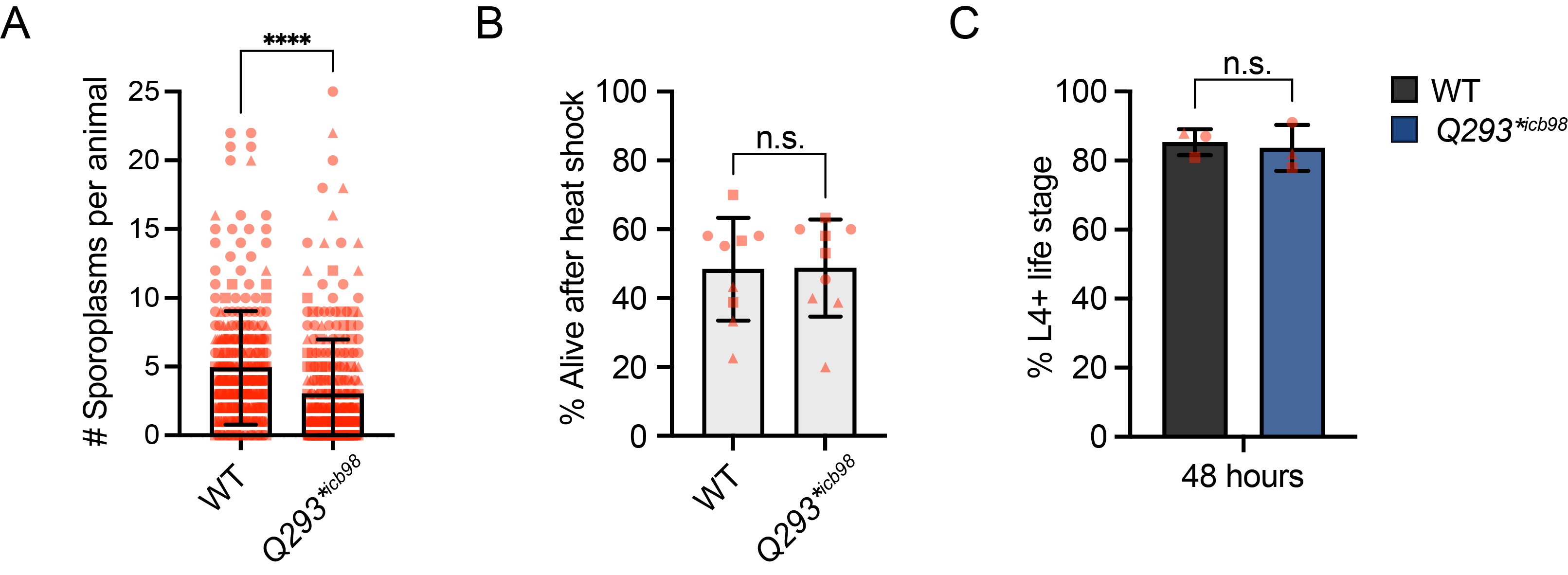

Supplement: S6 Fig — A) pals-25(Q293*)icb98 mutants exhibit resistance to N. parisii compared to WT at 3 hpi. **** p < 0.0001, Mann-Whitney test. n = 300 animals per genotype, three experimental replicates. Symbols represent the number of N. parisii sporoplasms infecting an individual animal, as determined by N. parisii-specific fluorescent FISH signal. B) pals-25(Q293*)icb98 mutants do not exhibit increased survival compared to WT after 2 h of heat shock at 37.5°C, followed by 24 h at 20°C. Unpaired t test. n = 9 plates, 30 animals per plate, tested in triplicate. C) pals-25(Q293*)icb98 mutants display similar development rate compared to WT. Unpaired t test. For A-C bar heights indicate mean values and error bars represent standard deviations. Different symbol shapes represent data points from assays performed on different days. Both the WT and pals-25(Q293*)icb98 mutants were tested in the icbIs4[chil-27p::gfp, col-12p::mCherry] transgenic background. (TIF) [file pgen.1010314.s006.tif]

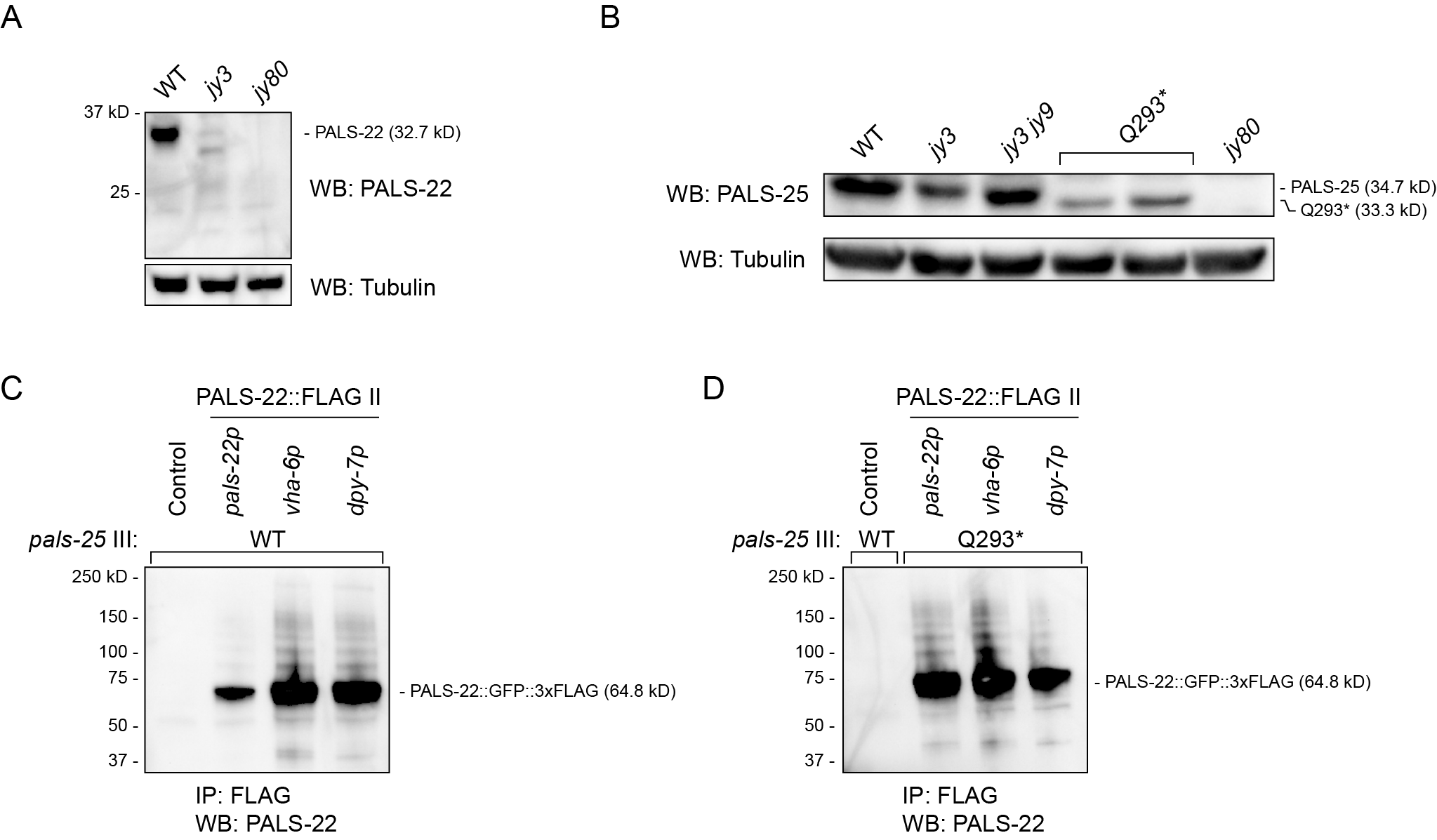

Supplement: S7 Fig — A) Custom antibodies raised against PALS-22 blot for protein in WT animals but not in pals-22(jy3) mutants or pals-22 pals-25(jy80) double mutants. B) Custom antibodies raised against PALS-25 blot for protein in WT and mutant strains analyzed in this study but not in pals-22 pals-25(jy80) double mutants. Note that pals-25(jy9) is phenotypically loss-of-function but the P38L substitution does not appear to alter PALS-25 abundance when compared to WT. C-D) Western blot analysis of PALS-22::GFP::3xFLAG expressed from the MosSCI locus on chromosome II using the endogenous promoter (pals-22p), an intestinal promoter (vha-6p), or epidermal promoter (dpy-7p). FLAG-IP successfully captures PALS-22 in both a WT pals-25 background (C) and in a pals-25(Q293*)jy111 mutant background (D). The GFP::3xFLAG control expressed from the MosSCI locus on chromosome II using the intestinal spp-5p promoter does not blot for PALS-22. Western blot was performed using anti-PALS-22 antibody. (TIF) [file pgen.1010314.s007.tif]

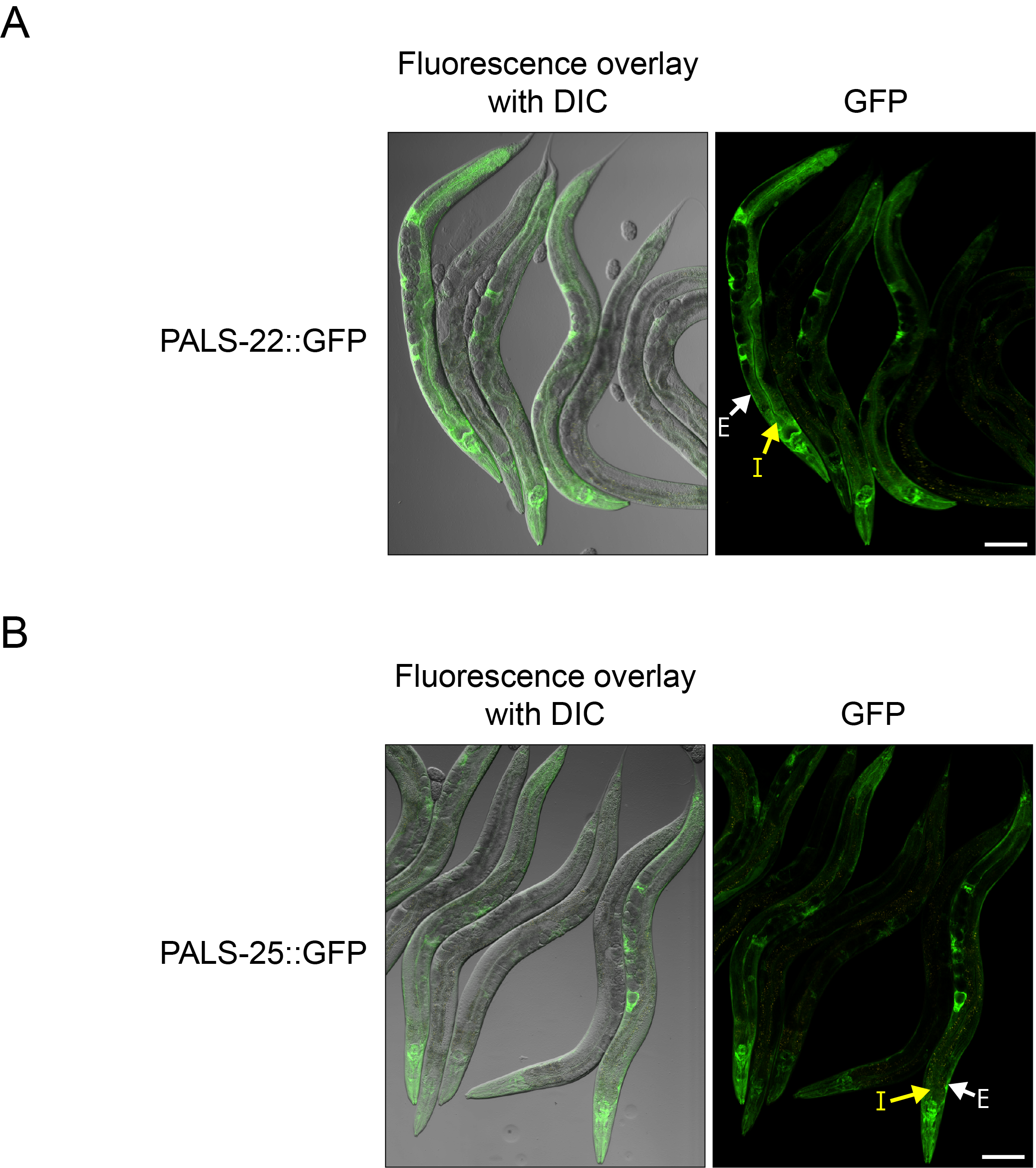

Supplement: S8 Fig — Confocal fluorescence images of adult animals carrying a multicopy TransgeneOme fosmid expressing PALS-22::GFP (A) or PALS-25::GFP (B). Fosmids have each gene tagged at the C terminus with GFP, surrounded by approximately 20 kb of endogenous regulatory region. Here, PALS-22 and PALS-25 are expressed in intestinal, epidermal, pharyngal and neuronal tissues. Yellow arrows indicate intestinal tissue (I) and white arrows indicate epidermal tissue (E). Scale bar = 100 μm. DIC = differential interference contrast. (TIF) [file pgen.1010314.s008.tif]

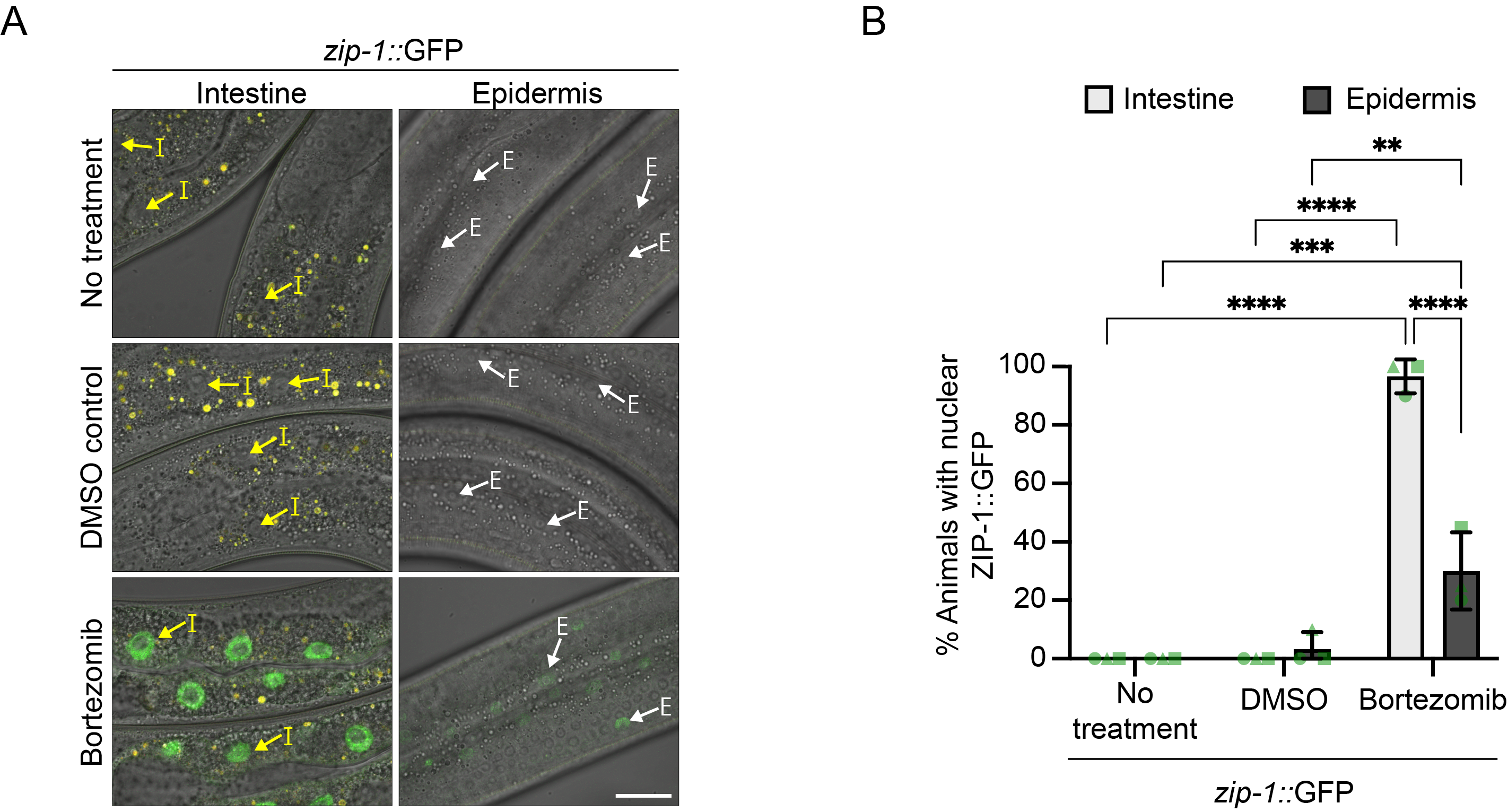

Supplement: S9 Fig — A) ZIP-1::GFP is expressed in intestinal and epidermal nuclei following bortezomib treatment, but not in untreated or DMSO control treated worms. Yellow arrows highlight intestinal nuclei ‘I’ and white arrows highlight epidermal nuclei ‘E’. Images are a composite of differential interference contrast, GFP, and RFP fluorescence channels and auto-fluorescent intestinal gut granules appear as yellow signal. Scale bar = 20 μm. B) Quantification of A, **** p < 0.0001, *** p < 0.001, ** p < 0.01, two-way ANOVA with Sidak’s multiple comparisons test. n = 3 experimental replicates, 20 animals per treatment and replicate assessed for both intestinal and epidermal ZIP-1::GFP expression for each replicate. Different symbols represent replicates performed on different days. Bar heights indicate mean values and error bars represent standard deviations. (TIF) [file pgen.1010314.s009.tif]

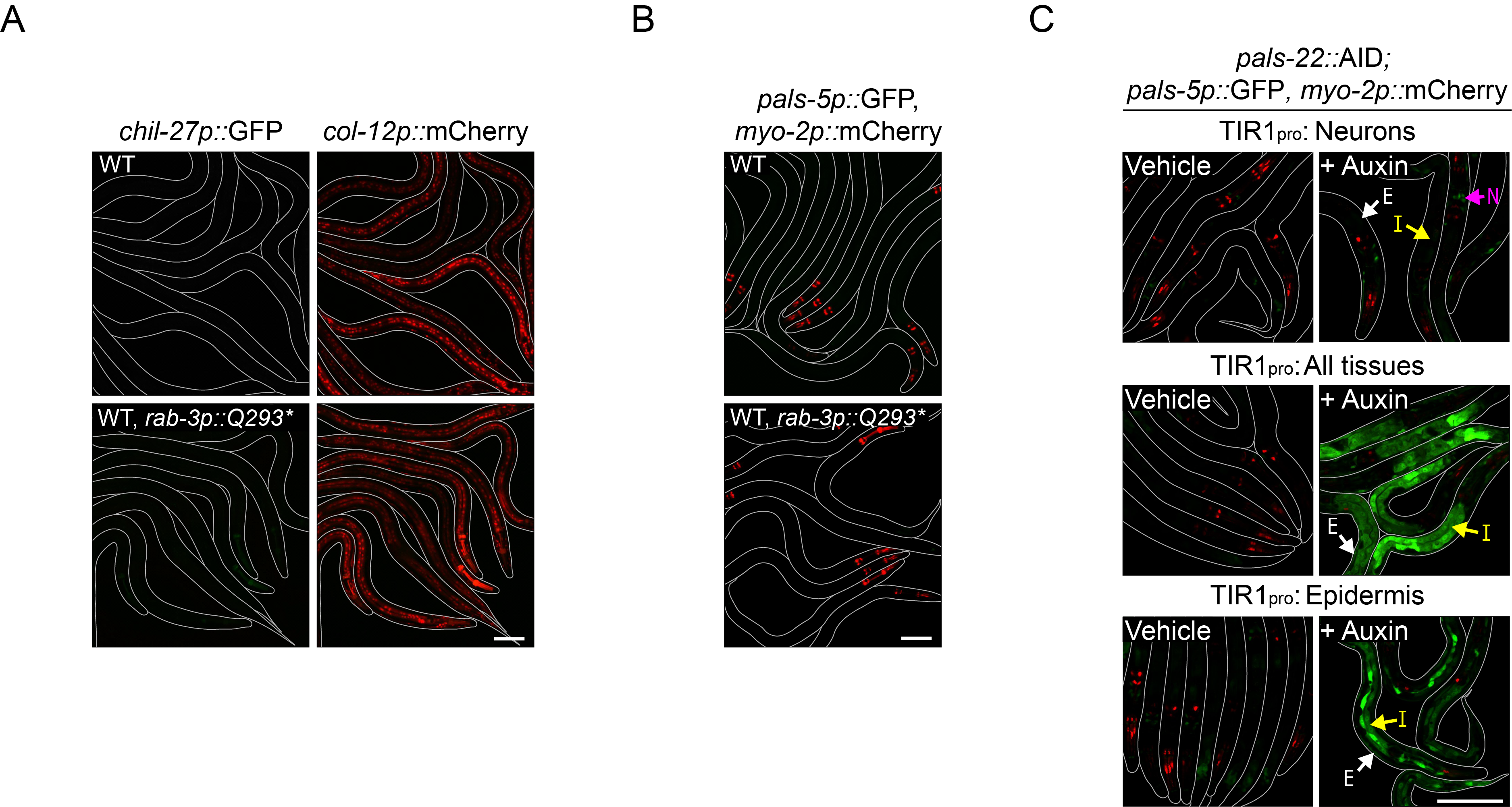

Supplement: S10 Fig — Neuronal (rab-3p)-specific expression of pals-25(Q293*) does not induce expression of the chil-27p::GFP ORR reporter (A) or the pals-5p::GFP IPR reporter (B). L4 animals are shown with lines denoting the outline of the body. Scale bar = 100 μm. C) Top, auxin-mediated depletion of PALS-22 in neurons induces pals-5p::GFP expression in neurons (magenta arrow ‘N’) but not in the epidermis (white arrow ‘E’) or intestine (yellow arrow ‘I’). Middle and bottom, depletion of PALS-22 in all tissues, or specifically in the epidermis, induces pals-5p::GFP expression in both the epidermis (white arrows ‘E’) and intestine (yellow arrows ‘I’). L1 animals treated with either auxin or vehicle control for 24 h at 20°C are shown. Scale bar = 50 μm. (TIF) [file pgen.1010314.s010.tif]

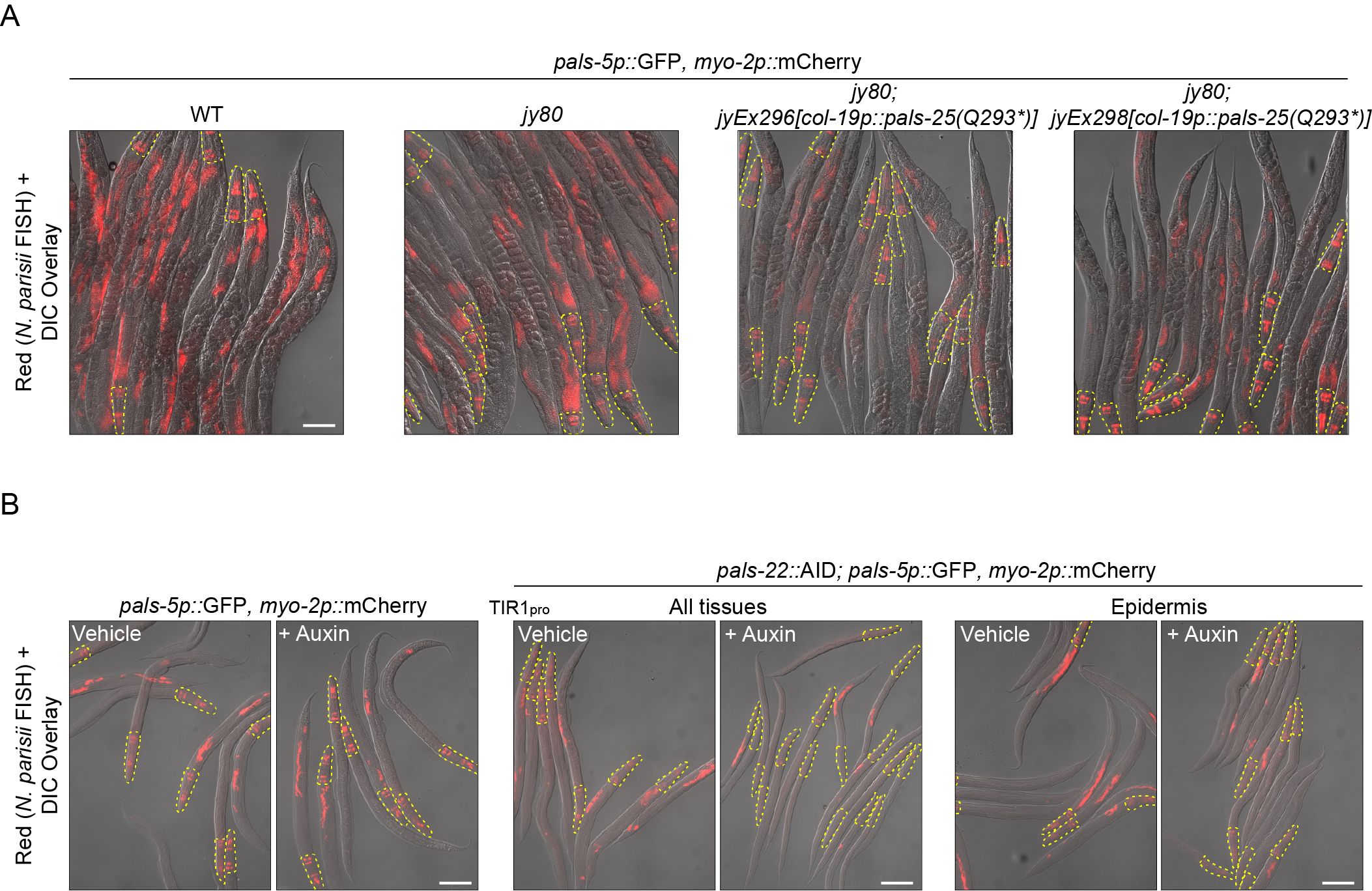

Supplement: S11 Fig — A) Representative images of WT, pals-22 pals-25(jy80), pals-22 pals-25(jy80); jyEx296[col-19p::pals-25(Q293*)], and pals-22 pals-25(jy80); jyEx298[col-19p::pals-25(Q293*)] animals infected as young adults with N. parisii, fixed at 30 hpi, and stained with N. parisii-specific FISH probe (red fluorescence). B) Representative images of WT animals, transgenic animals where PALS-22 can be ubiquitously depleted, or transgenic animals where PALS-22 is epidermally depleted, treated with either vehicle control or auxin. Control or auxin-treated strains were infected at L1 with N. parisii, fixed at 30 hpi, and stained with N. parisii-specific FISH probe (red fluorescence). For A, B scale bar = 100 μm. DIC = differential interference contrast. All strains are in a jyIs8[pals-5p::gfp, myo-2p::mCherry] background so head regions (yellow dashed lines) were omitted from pathogen load analysis due to expression of the myo-2p::mCherry co-injection marker. (TIF) [file pgen.1010314.s011.tif]
